# Energetic savings and cardiovascular dynamics of a marine euryhaline fish (*Myoxocephalus scorpius*) in reduced salinity

**DOI:** 10.1007/s00360-020-01336-8

**Published:** 2021-02-04

**Authors:** Erika Sundell, Daniel Morgenroth, Andreas Ekström, Jeroen Brijs, Michael Axelsson, Albin Gräns, Erik Sandblom

**Affiliations:** 1 Department of Biological and Environmental Sciences, University of Gothenburg, Gothenburg, Sweden; 2 Institute of Marine Biology, University of Hawai’i at Mānoa, Honolulu, USA; 3 Department of Animal Environment and Health, Swedish University of Agricultural Sciences, Gothenburg, Sweden

**Keywords:** Cardiovascular, Metabolic rate, Marine, Euryhaline, Salinity variability

## Abstract

Few studies have addressed how reduced water salinity affects cardiovascular and metabolic function in marine euryhaline fishes, despite its relevance for predicting impacts of natural salinity variations and ongoing climate change on marine fish populations. Here, shorthorn sculpin (*Myoxocephalus scorpius*) were subjected to different durations of reduced water salinity from 33 to 15 ppt. Routine metabolic rate decreased after short-term acclimation (4–9 days) to 15 ppt, which corresponded with similar reductions in cardiac output. Likewise, standard metabolic rate decreased after acute transition (3 h) from 33 to 15 ppt, suggesting a reduced energetic cost of osmoregulation at 15 ppt. Interestingly, gut blood flow remained unchanged across salinities, which contrasts with previous findings in freshwater euryhaline teleosts (e.g., rainbow trout) exposed to different salinities. Although plasma osmolality, [Na^+^], [Cl^−^] and [Ca^2+^] decreased in 15 ppt, there were no signs of cellular osmotic stress as plasma [K^+^], [hemoglobin] and hematocrit remained unchanged. Taken together, our data suggest that shorthorn sculpin are relatively weak plasma osmoregulators that apply a strategy whereby epithelial ion transport mechanisms are partially maintained across salinities, while plasma composition is allowed to fluctuate within certain ranges. This may have energetic benefits in environments where salinity naturally fluctuates, and could provide shorthorn sculpin with competitive advantages if salinity fluctuations intensify with climate change in the future.

**Supplementary Information:**

The online version contains supplementary material available at 10.1007/s00360-020-01336-8.

## Introduction

Many coastal marine environments are characterized by large fluctuations in water salinity, which challenges the osmotic homeostasis of animals inhabiting these environments. Moreover, alterations in environmental salinity are predicted to become more extreme in the future due to climate change resulting in, e.g., longer periods of exacerbated drought and evaporation, increased freshwater run-off due to altered precipitation patterns, and a more intensified mixing of water layers due to changing wind conditions (Durack et al. 2012; IPCC 2019; Zika et al. 2018). This will be particularly pronounced in shallow bays and estuaries, as these areas already experience pronounced variations in salinity (Bindoff et al. in press; Held and Soden 2006; IPCC 2013).

**Fig. 1:**
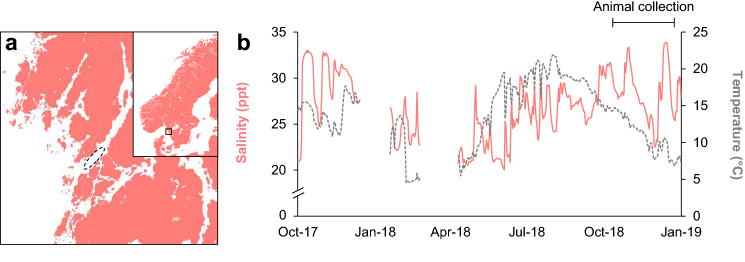
Geographical location and environmental characteristics of the Gullmarsfjorden area where shorthorn sculpin (*Myoxocephalus scorpius*) were collected. **a** The specific area from which shorthorn sculpin were caught within the Gullmarsfjorden (hatched ellipse) on the Swedish west coast (square within inset map). **b** Mean salinity (solid pink line) and temperature (hatched gray line) of the water body within Gullmarsfjorden measured at 6 m depth between October 2017 and January 2019 near to where shorthorn sculpin were caught (the period for animal collection is indicated by the horizontal bar in panel **b**). Logged data were unavailable during the two blank periods

Since environmental salinity impacts a wide range of biological processes and physiological functions, abrupt changes in salinity require aquatic animals such as marine fishes to either migrate to more favorable conditions, or to acclimate their physiology to maintain homeostasis (Kultz 2015). Consequently, salinity changes can lead to local alterations in species distribution, as well as changes in the structure of ecosystems (Smyth and Elliot 2016). Nonetheless, to assess how coastal marine fishes adjust to transient decreases in salinity, and to make predictions about future ecological implications of increased salinity variations, there is a need to understand the energetic consequences and underlying physiological responses governing salinity tolerance of fish. In this study, we used the shorthorn sculpin (*Myoxocephalus scorpius*) as a model to evaluate the effect of reduced salinity on cardiorespiratory function in a marine euryhaline teleost. The shorthorn sculpin is a relatively stationary demersal species that inhabits a variety of coastal habitats, including estuaries (Bone and Moore 2008; Coad and Reist 2004), and is known to tolerate salinities down to at least ~ 11.5 ppt (Foster 1969; Oikari 1978a, b). The population of shorthorn sculpin examined here were collected from an area on the Swedish west coast where the salinity regularly fluctuates between full-strength seawater of ~ 33 ppt and down to ~ 20 ppt within a few hours (Fig. 1).

Previous studies on other euryhaline fishes such as rainbow trout (*Oncorhynchus mykiss*) have shown that a transfer from freshwater to seawater involves an upregulation of active intestinal ion transport mechanisms and increased drinking rates to sustain the elevated demand for intestinal water absorption, which is necessary to compensate for osmotic water loss to the hypersaline environment (Brijs et al. 2015; Fuentes et al. 1997; Grosell 2013). This process involves increased intestinal epithelial Na^+^/K^+^-ATPase activity that creates a trans-epithelial Na^+^ gradient driving osmotic uptake of water into the blood (Grosell 2010; Whittamore 2012). Increased trans-epithelial transport of ions has been suggested to be reflected in an elevated standard metabolic rate (SMR), which is typically defined as the minimal aerobic energy required by an animal to maintain basal homeostatic processes (Chabot et al. 2016; Ern et al. 2014). Thus, the magnitude of the overall energy expenditure associated with osmoregulation should depend on the osmotic gradient between the fish and its environment, and thus theoretically, osmoregulation in water iso-osmotic to the blood plasma (i.e., ~ 10ppt) should be less energetically costly than both hyper- (i.e., seawater) and hypo-osmotic water (i.e., freshwater). Even so, empirical data are conflicting concerning the correlation between the energetic costs of osmoregulation and environmental salinity (Ern et al. 2014). From a metabolic point of view, this knowledge gap complicates predictions about the success of fishes in a future with more intense salinity fluctuations (Kultz 2015; Morgan and Iwama 1998).

The cardiovascular system drives tissue convection of water, ions, respiratory gases, nutrients, waste products and hormones (Sandblom and Gräns 2017). Changes in cardiovascular physiology are, therefore, crucial for coping with changing salinities. In rainbow trout, both acute exposure and chronic acclimation to increased salinity coincides with a suite of marked cardiovascular adjustments. Importantly, a twofold increase in gut blood flow occurs in seawater (Brijs et al. 2015, 2016a), which likely serves to increase the convective transport of absorbed ions and water away from the intestine to their respective sites for utilization or excretion (Brijs et al. 2015, 2016a; Farrell et al. 2009). The elevated gut blood flow is likely explained by a reduced gut vascular resistance (Sundell et al. 2018), and a sustained increase in cardiac output and stroke volume (Brijs et al. 2015, 2016a; Sundell et al. 2018). These responses are associated with a plastic restructuring of the heart, where the proportion of compact myocardium of the ventricle increases (Brijs et al. 2016b). Thus, while the cardiovascular responses of euryhaline fishes to increased salinity has been extensively characterized (Brijs et al. 2015, 2016a, b; Maxime et al. 1991; Morgenroth et al. 2019; Olson and Hoagland 2008; Sundell et al. 2018), virtually nothing is known about the cardiovascular mechanisms governing tolerance to reduced salinity in marine teleosts. Indeed, knowledge about the combined cardiovascular and metabolic responses would shed light onto how marine fishes cope with transient reductions in salinity, as well as potential physiological benefits and trade-offs when environmental salinity fluctuates.

In the present study, we measured routine metabolic rate (RMR) and cardiovascular responses (i.e., cardiac output, heart rate, stroke volume and gut blood flow) in shorthorn sculpin surgically instrumented with blood flow probes in full-strength seawater (33 ppt) and after short-term acclimation (i.e., 4–9 days) to reduced salinity (15 ppt). Moreover, in a separate group of uninstrumented sculpin, we characterized the dynamic changes in standard metabolic rate (SMR) over a five day period when acutely transferring from 33 to 15 ppt seawater. Specifically, we tested the hypotheses that: (i) the aerobic metabolic rate in sculpin is reduced, while plasma osmolality is maintained at salinities closer to iso-osmotic to the fish plasma (i.e., 15 ppt), and (ii) this is reflected in a reduced cardiac output and gastrointestinal blood perfusion.

## Methods

### Experimental animals

Shorthorn sculpin (*Myoxocephalus scorpius,* Linnaeus, 1758) were caught in baited traps by a local fisherman near Grundsund (Gullmarsfjorden) on the west coast of Sweden (Fig. 1). Sculpins were transported in aerated insulated cooler bins to the University of Gothenburg and held in a 1500 L tank (salinity: 33 ppt; temperature: 10 °C; 12:12 h light:dark photoperiod) for a minimum of 4 weeks prior to experiments to ensure acclimation to the new environment. The bottom of the tank was covered with gravel and half clay pots to supply suitable bottom substrates and dark hiding places for the fish. Fish were fed to satiation once a week with whole juvenile herring (*Clupea harengus*) and fasted for 6 days prior to experiments. All experimental protocols were approved by the ethical committee in Gothenburg (permit #165-2015).

### Experimental setup and experimental protocols

Two different experiments were performed as outlined below (see Table 1 for the biometrics of the experimental animals). In both experiments, fish were placed in respirometers (see below for details) submerged into a larger tank with recirculating water. The tanks were covered with black plastic and shielded by dark curtains to minimize visual disturbances. The water in the experimental tank was continuously aerated and maintained at 10 ± 1 °C.

**Table 1:** Morphological characteristics of Shorthorn sculpin (*Myoxocephalus scorpius*)

	Experiment 1		Experiment 2	
	33 ppt (*n* = 7)	15 ppt (*n* = 8)	33 ppt (*n* = 8–10)	15 ppt (*n* = 9–10)
Body mass (g)	135.2 ± 9.6	102.9 ± 12.3	245.6 ± 20.6	203.8 ± 16.8
Length (cm)	21.9 ± 4.4	20.1 ± 6.9	26.1 ± 6.2	24.9 ± 5.2

In experiment 1, sculpins were either kept in seawater (33 ppt) or subjected acutely to decreased salinity at 15 ppt. In experiment 2, sculpins either remained in seawater or were short-term acclimated (4–9 days) to 15 ppt salinity. Data are presented as means ± SEM

### Experiment 1: Metabolic and plasma homeostatic responses to reduced water salinity in uninstrumented sculpin

Fish were netted and placed into one of four custom-made PVC respirometers (3.1 L) and left undisturbed for at least 35 h before the experiments started. In this experiment, half of the fish were first kept in full-strength seawater (33 ppt) for the first 24 h (day 1), thereafter gradually exposed to reduced seawater salinity (i.e., 15 ppt) by the addition of freshwater over a 3-h period, and then continuously monitored for 4 days (i.e., days 2–5). The remaining fish served as controls and were treated identical but remained in seawater (i.e., 33 ppt) throughout the entire experimental protocol (i.e., 5 days in total). To standardize the experimental protocol, all steps involved in the procedure of changing the water salinity were performed for both groups after the first 24 h, with the exception that seawater instead of freshwater entered the tank for the control group. The oxygen consumption rate (MO_2_, a proxy for metabolic rate) was continuously recorded throughout the experimental protocol using intermittent flow respirometry (see details below). At the end of the experiment, fish were euthanized with a sharp blow to the head and a blood sample was immediately taken from the caudal vein using a heparinized syringe with a 23-gauge needle and analyzed as described below.

### Experiment 2: Cardiovascular and metabolic responses to short-term acclimation to reduced water salinity

In this experiment, half of the fish where acclimated to 15 ppt seawater for 4–9 days, while remaining fish where maintained in full-strength seawater (33 ppt) throughout the experiment. For surgical instrumentation, sculpins were anaesthetized in water corresponding to their acclimation salinity (i.e., 33 or 15 ppt) containing 100 mg l^−1^ of MS-222 (Tricaine methanesulfonate, Scanvacc, Hvam, Norway). The 15 ppt seawater was buffered with 200 mg l^−1^ of NaHCO_3_ (Merck, Darmstadt, Germany). Body mass and length were then determined before placing the sculpin on water-soaked foam on a surgical table. The gills were irrigated throughout the surgery with recirculating aerated seawater (33 or 15 ppt) at 10 °C containing 50 mg l^−1^ of MS-222. Again, the 15 ppt seawater was buffered with 100 mg l^−1^ of NaHCO_3._

**Fig. 2:**
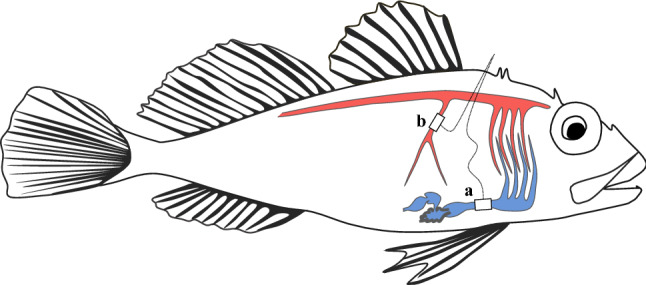
Schematic picture demonstrating the placement of flow probes on blood vessels in shorthorn sculpin (*Myoxocephalus scorpius*)*.* **a** Flow probes were placed around the ventral aorta to measure cardiac output, and **b** the celiacomesenteric artery to measure gut blood flow. Deoxygenated blood leaving the heart and entering the gills is represented by blue vasculature and oxygenated blood leaving the gills is represented by red vasculature

To record gut blood flow, a Transonic 1.5 PRS blood flow probe (Transonic systems, Inc, Ithaca, NY, USA) was placed around the celiacomesenteric artery, which branches from the dorsal aorta and is the main artery perfusing the stomach and intestine (Fig. 2; Seth and Axelsson 2009). The abdominal cavity was accessed via a ~ 1 cm lateral incision posterior to the pectoral fin. The celiacomesenteric artery was accessed by gently moving covering organs (*i.e.,* liver, spleen, stomach and gonads) using cotton free compressors, and dissected free from surrounding tissues using blunt forceps without damaging surrounding nerves or vessels (Gräns et al. 2013). The probe was placed around the exposed vessel and coupling gel (Surgilube, HR pharmaceuticals, Inc, York, USA) was added in the flow probe and the surrounding area to optimize the flow signal. The probe lead was secured with a 4–0 silk suture at the edge of the wound opening before closing the wound with interrupted 4–0 silk sutures. To measure cardiac output, a 2.5 PSL Transonic blood flow probe was placed around the ventral aorta. The fish was placed on its side with the operculum and the gill arches gently retracted to expose the opercular cavity. The ventral aorta, which in this species can be visibly identified underneath the tissue layers in the opercular cavity, was gently dissected free taking care not to damage surrounding nerves or vessels (Gräns et al. 2013; Seth and Axelsson 2009). The probe head was secured with two 4–0 silk sutures inside the opercular cavity, and the probe lead was secured with additional sutures along the side of the fish. The leads from both flow probes were then secured along the dorsal ridge, anterior to the dorsal fin (Fig. 2). The instrumented fish were then transferred into one of two identical custom-made polyethylene respirometers (6.1 L), and the flow probe leads were exited through an opening at the top of each respirometer. Fish were left undisturbed for at least 24 h at their acclimation salinity before cardiovascular and metabolic parameters were recorded for another 24 h (see Data acquisition and analyses for details). After the experiments, fish were euthanized with a sharp blow to the head.

### Data acquisition and calculations

#### Respirometry

The mass to volume ratios of the fish and the respirometers was 1:26 for experiment 1 and 1:27 for experiment 2, which is within the desirable ranges of 1:20–1:100 (Clark et al. 2013). Each respirometer was connected to an individual recirculation pump and a common flush pump (Eheim, Deizisau, Germany) through Tygon tubing. The recirculation pump inflow and outflow were connected diagonal on opposite sides of the respirometers to ensure a continuous recirculation and mixing of water inside the respirometer. A fiberoptic FireStingO_2_ system (FSO2-4, PyroScience, Aachen, Germany) continuously recorded the partial pressure of oxygen in the water within the respirometers. The oxygen optodes were two-point calibrated prior to each experiment in water maintained at 0% air saturation using sodium sulphite to remove oxygen from the water, and in fully oxygen-saturated water (100% air saturation) achieved by vigorous air-bubbling of the water. The flush pump was set to flush the respirometers with oxygen-saturated water for 10 min and was turned off for 15 min to measure the MO_2_ of the fish, which was determined from the decline in the partial pressure of oxygen in the respirometer water. Thus, each respirometer cycle was 25 min. When the respirometer was closed, the oxygen saturation never declined below 80%. Analog outputs from the FireStingO_2_ system were relayed to a 16SP PowerLab system connected to a computer with LabChart pro data acquisition software (7.3.2; ADInstruments, Castle Hill, Australia). The negative slopes in water oxygen saturation were used to calculate MO_2_ (mg O_2_ h^−1^) using Eq. 1:

1 $${\text{MO}}_{2} = \left( {\frac{{\left[{\left( {V_{r} - V_{f} } \right)*\Delta C_{{wO2}} } \right] }}{{\Delta t}}} \right) - \left( {\frac{{\left[ {V_{r} *\Delta C_{{wO2}} } \right] }}{{\Delta t}}} \right),$$

where *V*_r_ is the volume (*L*) of the respirometer, *V*_f_ is the volume (*L*) of the fish (derived from the body mass of the fish with the assumption that the fish density is 1 g ml of tissue^−1^), Δ*C*_wO2_ (% s^−1^) is the change in oxygen concentration in the water in the closed respirometer (calculated from the partial pressure of oxygen in the water taking salinity and temperature into account), and Δ*t* (s) is the time period during which Δ*C*_wO2_ was measured (Clark et al. 2013). Background (microbial) oxygen consumption was recorded for 2 h in empty respirometers after each experiment. This generated a minimum of four slopes used to calculate the mean background respiration, which was subtracted from the calculated MO_2_ to account for background respiration (Eq. 1; Svendsen et al. 2016). Since it took some time for the water to equilibrate in the respirometer after the flush pump was turned off, the Δ*C*_wO2_ was taken from the last 10 min of each closed cycle.

In experiment 1, the standard metabolic rate (SMR) was calculated from metabolic rate measurements during 24-h periods. In total, 50–60 metabolic rate measurements were obtained for each fish from each 24-h period, which were used to calculate the SMR during that 24-h period. If measurements of metabolic rate deviated more than two standard deviations from the 24 h mean, they were considered as outliers and excluded from the dataset. The SMR was determined as the mean of the lowest 10% of the remaining metabolic rate measurements (Clark et al. 2013; Svendsen et al. 2016). In experiment 2, metabolic rate measurements were obtained during a ~ 2–3 h period when the fish appeared calm and the cardiovascular recordings were stable (*see *Blood flow measurements below). Thus, 4–6 metabolic rate measurements were typically obtained from these periods, which were averaged to calculate routine metabolic rate (RMR) for each fish. Thus, we refer to the metabolic rate in experiment 2 as RMR rather than SMR as the metabolic rate data were obtained during a limited time interval and may have been affected by spontaneous activity (Fry 1971; Steffensen 2005).

#### Blood flow measurements

All Transonic flow probes were calibrated at 10 °C taking potential temperature effects on probe readings into account. The probes were connected to a Transonic flow meter (Transonic systems, Inc, Ithaca, NY, USA) and a 16SP PowerLab, which was connected to a computer with LabChart pro software. Heart rate was calculated in LabChart pro using the phasic cardiac output recording. Mean values for cardiac output, gut blood flow and heart rate were obtained during the 2–3-h period when metabolic rate measurements were taken. Stroke volume was calculated from cardiac output and heart rate according to Eq. 2, and the total tissue oxygen extraction (ml O_2_ ml blood^−1^) was estimated according to Eq. 3.

2 $${\text{Stroke volume}} = \frac{{\text{Cardiac output}}}{{\text{Heart rate}}},$$

3 $${\text{Tissue oxygen extraction}} = \frac{{{\text{RMR}}}}{{\text{Cardiac output}}}.$$

#### Analyses of hematological parameters and plasma composition

Blood samples were analyzed for hematocrit (%) and hemoglobin concentration ([hemoglobin]; mg ml^−1^). Hematocrit was determined as the fractional red cell volume after centrifugation of a sub-sample of blood in 80 μl heparinized microcapillary tubes at 10,000 rcf for 5 min in a hematocrit centrifuge (Haematokrit 210, Hettich, Tuttlingen, Germany). A handheld hemoglobin 201^+^ meter (Hemocue® AB, Ängelholm, Sweden) was used to determine hemoglobin concentration and the values were corrected for fish blood (Clark et al. 2008). Mean corpuscular hemoglobin concentration (MCHC, g dl^−1^) was calculated according to Eq. 4.

4 $${\text{MCHC = }}\frac{{\left[ {{\text{hemoglobin}}} \right]}}{{{\text{hematocrit}}}}*10.$$

The whole blood was then centrifuged at 10,000 rcf for 5 min in a microcentrifuge (Eppendorf® 5415D, Sigma-Aldrich Sweden AB, Stockholm, Sweden), and the plasma was collected and frozen at − 18 °C for later analyses of plasma ion composition. The concentrations (i.e., [X]) of Na^+^, Cl^−^, K^+^ and Ca^2+^ were determined using an ISE comfort Electrolyte Analyzer (Convergent technologies, Cölbe, Germany) and plasma osmolality was determined with a freezing point osmometer (Micro osmometer 3300, Advanced instruments, Norwood, USA). All blood and plasma analyses were performed in duplicates and averaged.

### Statistical analyses

Statistical analyses were performed using SPSS Statistics 22 (IBM Corp., Armonk, NY, USA). All data used were assessed to ensure that they did not violate the assumptions of the specific models outlined below. *F*-, *t*- and *P*-values obtained from the statistical analyses are reported throughout the text and all *P*-values < 0.05 were considered statistically significant. Unless otherwise specified, all data are presented as means ± S.E.M.

In experiment 1, an independent sample t-tests was used to compare blood hematocrit, [hemoglobin] and MCHC, as well as plasma [K^+^] and [Ca^2+^] between the two treatments (the 33 ppt and 15 ppt seawater). A Welch's t-test was used to compare plasma [Cl^−^] between treatments as this variable had a heterogenic distribution. For comparing plasma [Na^+^] between treatments, and in order to verify that the two experimental groups did not differ in SMR before the treatment was initiated (i.e., day 1 when both groups remained in 33 ppt water), a one-way ANCOVA with body mass as a covariate was used. Consequently, for the comparisons of SMR and plasma [Na^+^] between treatments all mean values were standardized, using body mass as a covariate, to an averaged-sized sculpin of 119 g. To compare SMR between the two treatments following the salinity change (*i.e.,* days 2–5), a linear mixed model was used. In the mixed model, individuals were set as subjects, time (days 2–5) as the repeated factor and body mass was included as a covariate. Time (days 2–5), treatment (the two salinities) and their interaction (i.e., time × treatment) were included as fixed factors in the model. An unstructured covariance matrix was used in the model as this provided the best fit to the data (i.e., the lowest Akaike’s Information Criterion). To meet the assumptions of the model, we applied a natural logarithm transformation on SMR.

In experiment 2, one-way ANCOVAs, using body mass as a covariate, were used to analyze differences between treatment groups for cardiac output, stroke volume, RMR and gut blood flow, as all of these parameters were affected by body mass. Consequently, for the comparisons, all mass-dependent variables were standardized to an averaged-sized sculpin (i.e., 225 g for cardiac output, stroke volume and RMR, and 219 g for gut blood flow). Independent sample t-tests were used to assess differences in heart rate and tissue oxygen extraction.

## Results

### Experiment 1—SMR and blood composition in shorthorn sculpin during acute exposure to reduced water salinity

**Fig. 3:**
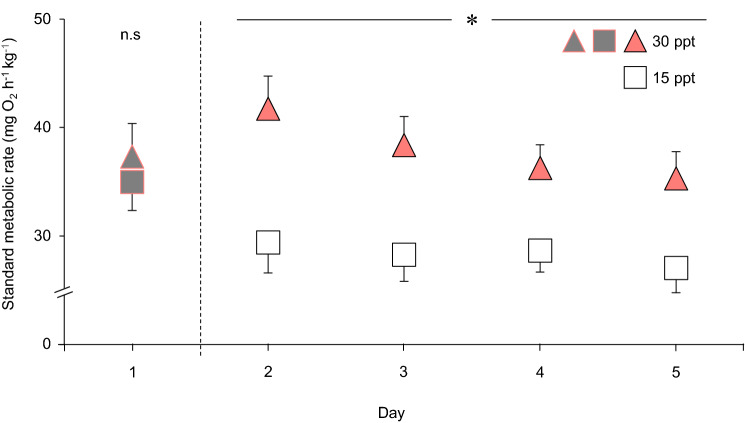
The effect of reduced salinity on standard metabolic rate (SMR) in uninstrumented shorthorn sculpin (*Myoxocephalus Scorpius*). All sculpins were initially kept at their 33 ppt seawater acclimation salinity (gray symbols, day 1). Sculpins were then either acutely exposed to a reduced water salinity of 15 ppt (open squares, *n* = 8; hatched line represents the salinity decrease) or maintained in 33 ppt seawater (pink triangles, *n* = 7) between days 2 and 5. For illustrative purposes, all mean values have been standardized, using body mass as a covariate, to an averaged-sized sculpin (i.e., 119 g). Asterisks (*) denote significant differences (*P* < 0.05) between treatments

Prior to the salinity change, the SMR at 33 ppt did not differ significantly between the two treatment groups (i.e., 37.3 ± 3.1 *c.f.,* 35.0 ± 2.6 mg O_2_ h^−1^ kg^−1^ in the subsequent 15 and 33 ppt groups, respectively; *F*_1, 11_ = 0.43, *P* = 0.526; Fig. 3). Following the salinity change, the SMR of fish exposed to 15 ppt was significantly reduced compared to fish remaining at 33 ppt, and was maintained reduced throughout the 4-day exposure period (*F*_1,11.5_ = 9.261, *P* = 0.015; Fig. 3). SMR significantly decreased throughout the 4-day exposure period (*F*_3,13_ = 3.535, P = 0.045), but no evidence was found of an interaction effect between time and treatment (*F*_3,13_ = 0.176, *P* = 0.911), suggesting that time spent in the respirometers affected both treatment groups similarly and was, therefore, unaffected by the water salinity.

At the end of the protocol (following the 4-day exposure, i.e., day 5), the plasma osmolality was significantly lower in sculpins exposed to 15 ppt seawater (343.7 ± 5.7 mOsm kg^−1^) compared to sculpins kept in 33 ppt seawater (403.6 ± 19.4 mOsm kg^−1^; *t*_8.2_ = 2.963, *P* = 0.018; Table 2). This was also reflected in lower plasma concentrations of Na^+^ (*F*_1,13_ = 27.238, *P* < 0.001), Cl^−^ (*t*_11.5_ = 5.458, *P* < 0.001) and Ca^2+^ (*t*_14_ = 2.199, *P* = 0.045), while the plasma concentration of K^+^ was unaltered (*t*_14_ = 1.932; *P* = 0.074; see Table 2). There were no significant differences in hemoglobin concentration, hematocrit or MCHC between treatment groups (Table 2).

**Table 2:** Effects of water salinity on hematological parameters in shorthorn sculpin (*Myoxocephalus scorpius)*

	33 ppt	15 ppt
Osmolality (mOsm kg^−1^)	403.6 ± 19.4	343.7 ± 5.7*
[Na^+^] (mmol l^−1^)	185.5 ± 4.8	148.4 ± 4.8*
[Cl^−^] (mmol l^−1^)	180.0 ± 6.4	139.3 ± 3.8*
[Ca^2+^] (mmol l^−1^)	0.72 ± 0.1	0.46 ± 0.1*
[K^+^] (mmol l^−1^)	3.5 ± 0.4	4.33 ± 0.2
Hb (mg ml^−1^)	50.6 ± 7.8	57.8 ± 8.7
Hct (% red blood cells)	16.0 ± 1.7	16.9 ± 2.6
MCHC (g dl^−1^)	30.3 ± 2.9	34.4 ± 2.1

Plasma osmolality, plasma ion concentrations, hemoglobin concentration (Hb), haematocrit (Hct), and mean corpuscular hemoglobin concentration (MCHC) in shorthorn sculpin kept in 33 ppt seawater or subjected to reduced water salinity (15 ppt) for 4 days. Data are presented as means ± SEM. Asterisks (*) denote significant differences (*P* ≤ 0.05) between treatment groups. Plasma concentration of Na^+^ was significantly affected by body mass, and mean values have, therefore, been standardized, using body mass as a covariate, to a averaged-sized sculpin of 119 g

### Experiment 2—cardiovascular and metabolic responses to short-term acclimation to reduced water salinity

**Fig. 4:**
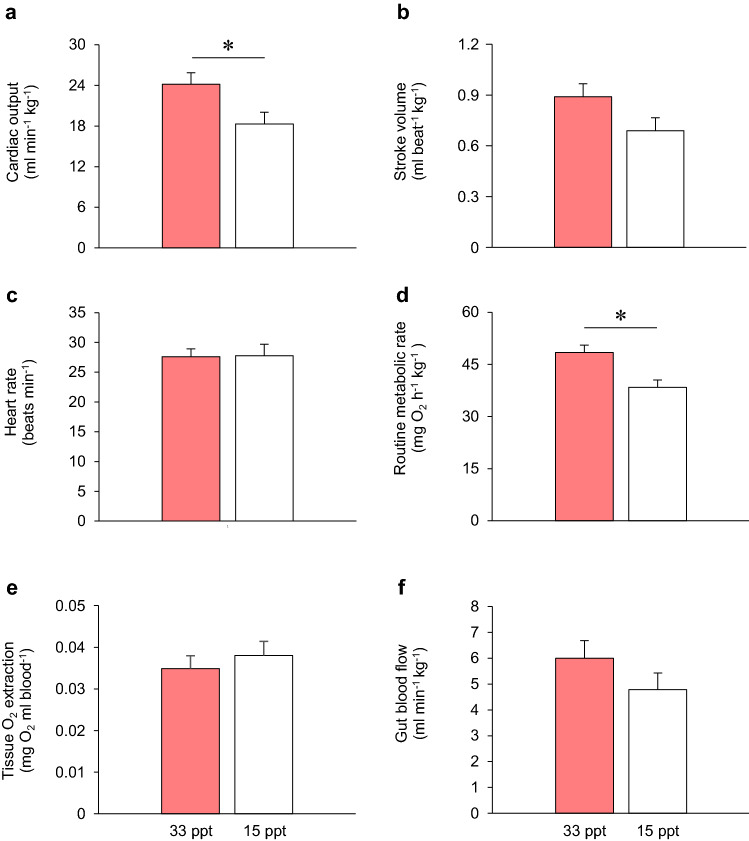
The effects of short-term acclimation to reduced salinity on routine cardiovascular and metabolic variables in shorthorn sculpin (*Myoxocephalus scorpius*)*.* Routine cardiorespiratory status of shorthorn sculpin acclimated to 33 ppt seawater (pink bars, *n* = 8–10) or 15 ppt seawater (open bars, *n* = 9–10) for 4–9 days. Panels **a** to **f** show cardiac output, stroke volume, heart rate, routine metabolic rate, tissue oxygen extraction and gut blood flow, respectively. All mass-dependent variables have been standardized, using body mass as a covariate, to an averaged-sized sculpin (i.e., 225 g for cardiac output, stroke volume and routine metabolic rate and 219 g for gut blood flow). All data are means ± SEM and asterisks (*) denote significant differences (*P* < 0.05) between treatments

The cardiac output of sculpin short-term acclimated to 15 ppt was significantly lower than that of sculpin acclimated to 33 ppt seawater (i.e., 18.3 ± 1.7 *c.f.* 24.2 ± 1.7 ml min^−1^ kg^−1^, respectively; *F*_1,17_ = 5.397, *P* = 0.033; Fig. 4a). This difference was likely explained by a reduced stroke volume in fish acclimated to 15 ppt compared to 33 ppt, although this was not statistically significant (i.e., 0.69 ± 0.08 *c.f.* 0.89 ± 0.08 ml beat^−1^ kg^−1^, respectively; *F*_1,17_ = 3.365, *P* = 0.084; Fig. 4b). Heart rate remained unchanged at 28 beats min^−1^ in both treatment groups (*t*_18_ = 0.58, *p* = 0.954; Fig. 4c). Interestingly, stroke volume was 22% lower in 15 ppt seawater, which was similar to the 24% reduction in cardiac output at the lower salinity. The effect of salinity on RMR was consistent with the reduction in cardiac output, as well as the general patterns in SMR observed in the uninstrumented sculpin (see Fig. 3). At 33 ppt, RMR was 48.4 ± 2.2 mg O_2_ h^−1^ kg^−1^ and decreased significantly to 38.3 ± 2.2 mg O_2_ h^−1^ kg^−1^ following short-term acclimation to 15 ppt seawater (*F*_1,17_ = 10.039, *P* = 0.006, for a 225 g fish; Fig. 4d). There were no significant effects of water salinity on the estimated tissue oxygen extraction (*t*_18_ = 0.690, *P* = 0.499; Fig. 4e) or gut blood flow (*F*_1,16_ = 1.659, *P* = 0.219; Fig. 4f).

## Discussion

The SMR of uninstrumented shorthorn sculpin in full-strength seawater (33 ppt) in the present study is consistent with previously reported values for this species under similar conditions (Sandblom et al. 2014). When subjected to a reduced water salinity of 15 ppt, the SMR of shorthorn sculpin decreased by ~ 23%, which could, at least in part, be explained by a reduced cost of osmoregulation at the lower salinity. Previous studies have demonstrated that the metabolic rate of rainbow trout were similar in freshwater and full-strength seawater (30–33 ppt; Brijs et al. 2015, 2017), but lower in iso-osmotic water (9 ppt; Altinok and Grizzle 2003). Taken together, this is consistent with the concept of a U-shaped relationship between metabolic rate and salinity (Costa 2009; Foster 1969). This is because increases in active ATP demanding transport mechanisms are required in both freshwater and seawater to counteract osmotic gradients between the external media and the internal environment of euryhaline fishes (Brijs et al. 2017; Ern et al. 2014; Evans et al. 2005; Kultz 2015; Marshall and Grosell 2006), while active transport mechanisms, such as intestinal solute-linked water absorption and branchial ion excretion are likely downregulated when the osmotic forces acting on the fish diminish at water salinities approaching iso-osmotic to the fish plasma (e.g., 15 ppt; Altinok and Grizzle 2003; Foster 1969). Since the costs of osmoregulation in dilute seawater are potentially reduced, sculpin may instead be able to allocate more energy into other important processes and activities such as feeding, growth and reproductive output (Bœuf and Payan 2001; Gaumet et al. 1995; Imsland et al. 2008; Liu et al. 2016).

Although the plasma osmolality was significantly lower in sculpins in 15 compared to 33 ppt seawater, these values were still well within the normal range for teleost fishes (Nordlie 2009). The decreased plasma osmolality at 15 ppt was also reflected by reduced plasma [Na^+^], [Cl^−^] and [Ca^2+^]. This indicates that shorthorn sculpin do not entirely downregulate ion excretion processes to closely maintain plasma composition in the lower environmental osmolality at 15 ppt. Yet, there were no clear signs of cellular osmotic stress at 15 ppt, as hematocrit, [hemoglobin] and plasma [K^+^] were similar across salinities (Soegianto et al. 2017). These latter observations suggest that shorthorn sculpin are relatively weak plasma osmoregulators, and may instead regulate cellular osmolality and ion composition strictly while allowing plasma osmolality and ion composition to fluctuate with the surrounding water within certain ranges.

The abovementioned reduction in SMR of uninstrumented shorthorn sculpin acutely transferred to 15 ppt in experiment 1 was largely consistent with the 21% reduction in RMR, as well as the 24% reduction in cardiac output, in instrumented sculpin short-term acclimated to 15 ppt in experiment 2. The reduced blood flow is not surprising, as fish like all other vertebrates, adjust tissue blood perfusion and/or oxygen extraction in accordance with oxygen demands (Farrell et al. 2009; Steinhausen et al. 2008). However, the present study shows that circulatory oxygen convection is modulated in response to different metabolic needs at different salinities in shorthorn sculpin, while the estimated tissue oxygen extraction remained unaltered. Moreover, the decrease in cardiac output of sculpin appeared to be caused by a reduction in stroke volume, as heart rate remained unchanged. The modulation of cardiac output via stroke volume in relation to environmental salinity is consistent with previously observed responses in rainbow trout, and may thus be a general feature of teleosts coping with changing salinities (Brijs et al. 2016a, b; Maxime et al. 1991; Morgenroth et al. 2019; Sundell et al. 2018). It is possible that shifting the baseline cardiac output through changes in stroke volume at different salinities is beneficial as it allows fish to maintain a high heart rate scope for situations requiring more rapid adjustments in oxygen convection, such as when escaping predators or digesting a meal (Brijs et al. 2015; Farrell et al. 2009; Seth and Axelsson 2009).

Previous studies have demonstrated a substantially increased gut blood flow in euryhaline rainbow trout when both acutely and chronically transitioning from freshwater to seawater (Brijs et al. 2015, 2016a). For these fish, an increased gut blood flow is likely necessary to increase intestinal water absorption and to transport absorbed ions and water away from the gut to their respective sites for utilization or excretion (Brijs et al. 2015, 2016a). While we hypothesized that exposure to conditions approaching iso-osmotic (i.e., 15 ppt) would result in reduced intestinal water absorption and gut blood flow compared to sculpin in 33 ppt seawater, the gut blood flow of shorthorn sculpin remained largely similar across salinities. Importantly, however, 15 ppt still reflects a hyperosmotic environment, and so the sculpin would still need to hypo-osmoregulate to maintain osmotic homeostasis (Foster 1969). This involves drinking to counteract water loss and sufficient circulatory perfusion of the gut for osmoregulatory purposes. While this study did not measure drinking rates, one possible reason for the similar gut blood flow across salinities is that the drinking rate was maintained in sculpin subjected to the reduced water salinity, which could require a sustained gut blood flow. Indeed, this is consistent with the lower plasma osmolality observed in fish at 15 ppt compared to 33 ppt.

As gut blood flow was not responsible, another potential explanation for the overall reduction in cardiac output in 15 ppt seawater is that there were changes to the intra-branchial blood flow distribution with a diminished gill blood flow for osmoregulatory purposes. In the arterio-venous pathway of the branchial circulation, which is the primary pathway where branchial osmoregulation takes place, blood is shunted from the arterio-arterial pathway via arterio-venous anastomoses (Costa 2009; Olson 2002). Thus, it could be speculated that less arterio-venous shunting is required at salinities closer to iso-osmotic to the fish plasma due to a decreased need for active osmoregulation at the gills (Evans 1993, 2008; Greenwell et al. 2003). This would be consistent with the diminished cardiac output and metabolic rate observed in shorthorn sculpin short-term acclimated to 15 ppt salinity in the present study. Measurements of arterio-venous branchial blood flow in fishes at different salinities, therefore, represent an interesting area for research.

In conclusion, this is the first study to show the concomitant metabolic and cardiovascular responses to decreased salinity in a euryhaline marine teleost. From an ecological and conservation perspective, such physiological information is crucial to understand and predict how coastal and estuarine fishes adjust to natural salinity variations, as well as intensified salinity fluctuations in the future. We show that shorthorn sculpin exposed to short-term reductions in salinity experience reductions in osmoregulatory costs and cardiac output, which are likely linked to a reduced need for active epithelial transport mechanisms in lower salinity waters. It is reasonable to speculate that the downregulation of epithelial ion transport occurs at several osmoregulatory sites, which combined contribute towards the overall decrease in metabolic activity and circulatory convection. Furthermore, our data suggest that since shorthorn sculpin inhabits environments that may exhibit frequent and relatively large salinity fluctuations, an osmoregulatory strategy where plasma osmolality and ion composition are allowed to fluctuate within certain ranges may provide an ‘energy-saving’ function (Kirsch and Meister 1982). While a life-history that involves distinct migration events from freshwater to seawater, such as in some salmonids, may require more complete acclimatization for long-term survival and performance in the marine environment, fishes adapted to marine coastal habitats such as the shorthorn sculpin may not require such acclimatization but simply a capacity to “endure” short-term, transient salinity changes (Edwards and Marshall 2013; Kultz 2015; Marshall 2013). Since such complete acclimatization can be expected to be slower and more energetically costly, “endurance strategies” as observed here in shorthorn sculpin may provide significant energetic benefits in highly variable environments and when salinity changes are transient.

## Supplementary Information

Below is the link to the electronic supplementary material.

Supplementary file1 (PDF 342 KB)

## Data Availability

All data generated or analyzed during this study are included in this published article [and its supplementary information files].
