# Supplementary material for: Energetic savings and cardiovascular dynamics of a marine euryhaline fish (Myoxocephalus scorpius) in reduced salinity
Source: J Comp Physiol B. 2021 Feb 4;191(2):301–11. doi: 10.1007/s00360-020-01336-8 (PMC7895773; doi:10.1007/s00360-020-01336-8)
Supplement: Supplementary file 1 — Supplementary file1 (PDF 342 KB) [file 360_2020_1336_MOESM1_ESM.pdf]

Supplementary information for:

Energetic and cardiovascular dynamics of a marine euryhaline fish (*Myoxocephalus scorpius*) in reduced salinity.

Journal of Comparative Physiology B

Sundell E,* Morgenroth D, Ekström A, Brijs J, Axelsson M, Gräns A, Sandblom E.

erika.sundell@hotmail.com

This PDF includes datasets S1 to S4.

Dataset S1

Salinity and temperature data for the period 2017-10-01 to 2019-01-01.

Salinity and temperature was measured at 6 meters depth at Kristineberg marine research station. No data was available during the two blank periods. Data was obtained from: https://www.weather.loven.gu.se/kristineberg/en/data.shtml

Date	Salinity (ppt)	Temperature (degC)
01-10-2017	21.00	14.40
02-10-2017	20.92	14.72
03-10-2017	21.01	14.82
04-10-2017	21.31	14.39
05-10-2017	23.27	14.39
06-10-2017	31.43	15.45
07-10-2017	32.12	15.47
08-10-2017	32.37	15.45
09-10-2017	32.59	15.43
10-10-2017	32.78	15.46
11-10-2017	32.96	15.52
12-10-2017	32.76	15.39
13-10-2017	33.02	15.29
14-10-2017	32.81	15.16
15-10-2017	32.80	15.04
16-10-2017	32.65	14.87
17-10-2017	32.58	14.76
18-10-2017	32.20	14.36
19-10-2017	31.30	14.12
20-10-2017	28.56	12.99
21-10-2017	27.39	12.70
22-10-2017	27.17	12.55
23-10-2017	26.78	12.34
24-10-2017	26.68	11.85
25-10-2017	25.47	11.94
26-10-2017	24.83	12.26
27-10-2017	25.02	12.22
28-10-2017	25.37	12.12
29-10-2017	27.77	12.54
30-10-2017	32.23	13.94
31-10-2017	32.83	14.18
01-11-2017	32.43	14.11
02-11-2017	32.39	13.74
03-11-2017	32.74	13.84

04-11-2017	32.58	13.66
05-11-2017	32.55	13.27
06-11-2017	32.12	12.68
07-11-2017	30.53	12.27
08-11-2017	29.12	11.79
09-11-2017	28.34	11.54
10-11-2017	27.86	11.39
11-11-2017	27.68	10.86
12-11-2017	28.94	11.59
13-11-2017	31.67	12.79
14-11-2017	31.95	12.78
15-11-2017	30.98	11.98
16-11-2017	30.70	11.56
17-11-2017	30.68	11.55
18-11-2017	30.47	11.17
19-11-2017	30.19	10.90
20-11-2017	30.63	11.17
21-11-2017	31.16	11.48
22-11-2017	31.53	12.70
23-11-2017	31.26	14.31
24-11-2017	31.04	15.79
25-11-2017	31.03	16.96
26-11-2017	31.02	17.59
27-11-2017	30.98	17.71
28-11-2017	30.86	17.17
29-11-2017	30.72	16.80
30-11-2017	30.49	16.72
01-12-2017	30.30	16.75
02-12-2017	30.23	16.65
03-12-2017	30.13	16.45
04-12-2017	29.71	15.68
05-12-2017	28.76	15.39
06-12-2017	28.30	15.16
07-12-2017	27.81	15.74
08-12-2017	27.63	16.71
09-12-2017	27.50	16.70
10-12-2017	27.31	15.98
11-12-2017		15.41
12-12-2017		15.23
13-12-2017		15.62
14-12-2017		15.75
15-12-2017		
16-12-2017		
17-12-2017		
18-12-2017		
19-12-2017		
20-12-2017		
21-12-2017		
22-12-2017		
23-12-2017		
24-12-2017		
25-12-2017		
26-12-2017		
27-12-2017		
28-12-2017		
29-12-2017		
30-12-2017		
31-12-2017		

01-01-2018		
02-01-2018		
03-01-2018		
04-01-2018		
05-01-2018		
06-01-2018		
07-01-2018		
08-01-2018		
09-01-2018		
10-01-2018		
11-01-2018		
12-01-2018		
13-01-2018		
14-01-2018		
15-01-2018		
16-01-2018		
17-01-2018		
18-01-2018		
19-01-2018	26.79	8.44
20-01-2018	26.64	10.69
21-01-2018	26.07	11.71
22-01-2018	25.20	12.23
23-01-2018	23.97	11.37
24-01-2018	22.58	9.26
25-01-2018	22.35	11.68
26-01-2018	22.25	12.62
27-01-2018	22.14	13.10
28-01-2018	22.14	13.32
29-01-2018	22.43	13.39
30-01-2018	22.61	13.56
31-01-2018	23.36	13.67
01-02-2018	24.60	13.62
02-02-2018	25.10	13.18
03-02-2018	25.24	12.94
04-02-2018	26.33	12.45
05-02-2018	27.66	11.84
06-02-2018	27.98	8.84
07-02-2018	25.92	4.50
08-02-2018	25.72	4.67
09-02-2018	25.47	4.74
10-02-2018	23.41	4.65
11-02-2018	22.77	4.55
12-02-2018	22.73	4.54
13-02-2018	22.83	4.58
14-02-2018	23.80	4.76
15-02-2018	23.84	4.80
16-02-2018	23.95	4.80
17-02-2018	23.61	4.80
18-02-2018	23.90	4.91
19-02-2018	24.81	5.03
20-02-2018	28.48	5.82
21-02-2018	24.34	4.92
22-02-2018	22.95	4.85
23-02-2018	22.72	5.10
24-02-2018		
25-02-2018		
26-02-2018		
27-02-2018		

28-02-2018		
01-03-2018		
02-03-2018		
03-03-2018		
04-03-2018		
05-03-2018		
06-03-2018		
07-03-2018		
08-03-2018		
09-03-2018		
10-03-2018		
11-03-2018		
12-03-2018		
13-03-2018		
14-03-2018		
15-03-2018		
16-03-2018		
17-03-2018		
18-03-2018		
19-03-2018		
20-03-2018		
21-03-2018		
22-03-2018		
23-03-2018		
24-03-2018		
25-03-2018		
26-03-2018		
27-03-2018		
28-03-2018		
29-03-2018		
30-03-2018		
31-03-2018		
01-04-2018		
02-04-2018		
03-04-2018		
04-04-2018		
05-04-2018		
06-04-2018		
07-04-2018		
08-04-2018		
09-04-2018		
10-04-2018	20.72	8.15
11-04-2018	21.48	8.14
12-04-2018	22.15	6.14
13-04-2018	22.27	5.47
14-04-2018	22.50	5.89
15-04-2018	21.86	6.45
16-04-2018	20.75	7.02
17-04-2018	21.19	6.40
18-04-2018	20.86	6.80
19-04-2018	20.91	7.11
20-04-2018	20.68	7.39
21-04-2018	20.53	7.81
22-04-2018	20.65	7.92
23-04-2018	20.96	7.63
24-04-2018	20.90	7.69
25-04-2018	20.87	7.88
26-04-2018	20.98	8.11

27-04-2018	21.08	8.40
28-04-2018	21.11	8.66
29-04-2018	24.01	8.39
30-04-2018	27.53	8.09
01-05-2018	29.20	7.96
02-05-2018	25.51	8.70
03-05-2018	25.70	9.12
04-05-2018	25.01	9.56
05-05-2018	25.05	9.68
06-05-2018	25.18	9.75
07-05-2018	24.94	10.59
08-05-2018	24.41	11.26
09-05-2018	23.90	11.81
10-05-2018	23.40	12.31
11-05-2018	22.04	13.31
12-05-2018	21.80	13.40
13-05-2018	22.60	13.06
14-05-2018	23.26	12.35
15-05-2018	22.64	13.25
16-05-2018	22.18	13.78
17-05-2018	23.48	12.45
18-05-2018	25.19	11.01
19-05-2018	23.85	12.33
20-05-2018	22.33	14.90
21-05-2018	21.19	15.64
22-05-2018	20.91	15.96
23-05-2018	20.90	16.32
24-05-2018	21.12	16.33
25-05-2018	21.17	16.57
26-05-2018	21.22	16.84
27-05-2018	21.32	16.85
28-05-2018	21.26	17.34
29-05-2018	21.22	17.51
30-05-2018	21.19	17.87
31-05-2018	21.19	18.35
01-06-2018	20.87	18.79
02-06-2018	20.37	19.14
03-06-2018	20.23	19.45
04-06-2018	20.04	19.54
05-06-2018	25.31	13.22
06-06-2018	24.90	14.02
07-06-2018	22.14	17.39
08-06-2018	21.43	18.02
09-06-2018	21.54	18.05
10-06-2018	21.74	17.99
11-06-2018	20.84	18.84
12-06-2018	25.98	15.73
13-06-2018	27.44	15.22
14-06-2018	24.53	17.51
15-06-2018	25.50	17.59
16-06-2018	24.65	17.53
17-06-2018	25.03	17.71
18-06-2018	25.29	17.59
19-06-2018	25.61	17.11
20-06-2018	25.33	16.55
21-06-2018	25.06	16.82
22-06-2018	27.78	15.83
23-06-2018	30.22	14.50

24-06-2018	30.05	14.77
25-06-2018	29.67	15.21
26-06-2018	29.04	15.96
27-06-2018	28.40	16.98
28-06-2018	27.39	18.55
29-06-2018	26.64	19.24
30-06-2018	28.39	17.60
01-07-2018	28.39	17.52
02-07-2018	27.82	18.31
03-07-2018	26.93	19.23
04-07-2018	26.07	19.55
05-07-2018	25.96	19.68
06-07-2018	24.92	19.88
07-07-2018	25.95	19.66
08-07-2018	26.92	19.40
09-07-2018	26.05	20.13
10-07-2018	29.17	17.11
11-07-2018	27.06	19.59
12-07-2018	25.56	20.64
13-07-2018	24.95	21.02
14-07-2018	23.42	21.19
15-07-2018	23.26	21.01
16-07-2018	24.68	20.78
17-07-2018	28.92	17.41
18-07-2018	30.17	15.96
19-07-2018	26.93	19.64
20-07-2018	25.77	20.57
21-07-2018	25.59	20.70
22-07-2018	25.98	20.09
23-07-2018	26.05	20.04
24-07-2018	26.94	19.60
25-07-2018	29.22	18.52
26-07-2018	29.26	18.79
27-07-2018	29.09	19.26
28-07-2018	29.19	19.35
29-07-2018	26.92	21.03
30-07-2018	25.55	21.37
31-07-2018	25.06	21.71
01-08-2018	24.35	21.88
02-08-2018	24.23	21.88
03-08-2018	24.18	21.68
04-08-2018	24.16	21.71
05-08-2018	24.25	21.62
06-08-2018	25.80	20.74
07-08-2018	27.38	20.02
08-08-2018	28.93	19.24
09-08-2018	27.43	20.03
10-08-2018	29.49	18.89
11-08-2018	29.33	18.74
12-08-2018	28.23	19.16
13-08-2018	28.80	19.15
14-08-2018	29.47	19.04
15-08-2018	29.43	19.12
16-08-2018	28.75	19.30
17-08-2018	28.33	19.17
18-08-2018	27.94	19.12
19-08-2018	28.01	18.87
20-08-2018	27.96	18.87

21-08-2018	28.13	18.85
22-08-2018	27.73	18.95
23-08-2018	26.61	18.89
24-08-2018	26.95	18.88
25-08-2018	26.89	18.60
26-08-2018	27.06	18.61
27-08-2018	27.07	18.30
28-08-2018	27.20	18.35
29-08-2018	27.42	18.49
30-08-2018	27.30	18.44
31-08-2018	27.13	18.27
01-09-2018	27.54	18.19
02-09-2018	27.48	18.32
03-09-2018	27.87	18.51
04-09-2018	27.21	18.60
05-09-2018	27.06	18.74
06-09-2018	26.71	18.78
07-09-2018	26.38	18.70
08-09-2018	26.34	18.51
09-09-2018	24.97	18.31
10-09-2018	24.92	18.38
11-09-2018	25.32	18.26
12-09-2018	25.49	17.84
13-09-2018	25.68	17.50
14-09-2018	25.64	17.32
15-09-2018	25.69	17.08
16-09-2018	25.74	16.72
17-09-2018	26.42	16.78
18-09-2018	26.56	16.70
19-09-2018	27.17	16.94
20-09-2018	27.31	17.03
21-09-2018	27.48	16.69
22-09-2018	28.25	16.23
23-09-2018	27.10	15.88
24-09-2018	27.07	15.66
25-09-2018	29.51	15.91
26-09-2018	31.42	15.92
27-09-2018	31.19	15.33
28-09-2018	30.28	14.85
29-09-2018	30.32	15.04
30-09-2018	31.34	15.11
01-10-2018	30.79	14.67
02-10-2018	30.36	14.59
03-10-2018	29.96	14.09
04-10-2018	31.05	14.73
05-10-2018	31.52	14.99
06-10-2018	31.31	14.55
07-10-2018	31.35	14.61
08-10-2018	31.71	14.75
09-10-2018	31.57	14.52
10-10-2018	31.02	14.27
11-10-2018	30.26	14.33
12-10-2018	29.80	14.32
13-10-2018	29.14	14.48
14-10-2018	28.79	14.74
15-10-2018	28.62	14.61
16-10-2018	28.41	14.52
17-10-2018	28.34	14.55

18-10-2018	27.71	14.27
19-10-2018	28.04	14.33
20-10-2018	28.02	14.16
21-10-2018	28.02	14.10
22-10-2018	28.34	13.97
23-10-2018	28.51	13.63
24-10-2018	28.14	13.17
25-10-2018	28.30	13.28
26-10-2018	31.63	14.04
27-10-2018	30.60	13.41
28-10-2018	31.89	13.38
29-10-2018	32.98	13.55
30-10-2018	33.29	13.34
31-10-2018	33.32	12.95
01-11-2018	31.93	11.99
02-11-2018	31.42	12.16
03-11-2018	30.74	12.36
04-11-2018	30.03	12.42
05-11-2018	29.24	12.37
06-11-2018	29.21	12.12
07-11-2018	29.11	11.99
08-11-2018	28.89	11.99
09-11-2018	28.45	11.94
10-11-2018	28.28	11.69
11-11-2018	27.98	11.54
12-11-2018	27.59	11.58
13-11-2018	27.27	11.56
14-11-2018	27.18	11.61
15-11-2018	27.24	11.73
16-11-2018	27.34	11.55
17-11-2018	27.24	11.30
18-11-2018	26.88	11.25
19-11-2018	26.77	11.20
20-11-2018	26.26	10.93
21-11-2018	26.04	10.47
22-11-2018	25.50	9.71
23-11-2018	24.99	9.51
24-11-2018	24.48	8.82
25-11-2018	25.61	9.63
26-11-2018	26.02	9.93
27-11-2018	25.85	9.84
28-11-2018	25.38	9.04
29-11-2018	24.53	7.83
30-11-2018	22.77	7.66
01-12-2018	22.49	7.79
02-12-2018	22.70	7.89
03-12-2018	23.30	8.11
04-12-2018	23.11	8.13
05-12-2018	26.97	9.18
06-12-2018	29.51	10.15
07-12-2018	29.52	10.05
08-12-2018	27.60	9.06
09-12-2018	27.74	8.86
10-12-2018	30.06	9.49
11-12-2018	32.88	10.53
12-12-2018	33.66	10.62
13-12-2018	33.80	10.43
14-12-2018	33.86	10.40

15-12-2018	33.90	10.34
16-12-2018	33.24	9.54
17-12-2018	32.21	8.09
18-12-2018	31.50	7.41
19-12-2018	29.87	7.28
20-12-2018	28.20	7.59
21-12-2018	26.74	7.61
22-12-2018	26.16	7.41
23-12-2018	25.76	7.17
24-12-2018	25.49	6.85
25-12-2018	25.36	6.87
26-12-2018	26.37	7.27
27-12-2018	26.34	7.32
28-12-2018	29.79	7.92
29-12-2018	29.42	7.73
30-12-2018	30.16	8.09
31-12-2018	30.06	8.10
01-01-2019	27.89	7.32

Dataset S2

Standard metabolic rate (SMR) data for uninstrumented shorthorn sculpin.

SMR (mg O_2 h^{-1}) is displayed before any treatment at 33 ppt salinity (before) and at days 1-4 following exposure to either diluted seawater (15 ppt) for the treatment group or full-strength seawater (33 ppt) for the control group.

		**Before**	**Day 1**	**Day 2**	**Day 3**	**Day 4**
**Group**	**ID**	**SMR**	**SMR**	**SMR**	**SMR**	**SMR**
control	1	2.45	2.62	2.18	2.42	2.61
control	2	3.90	3.57	3.71	3.80	3.62
control	3	7.29	9.08	8.15	6.76	5.98
control	4	6.07	6.02	5.87	5.94	5.55
control	5	6.72	6.92	6.17	5.67	5.27
control	7	5.69	5.76	4.96	5.21	5.78
control	8	5.57	5.32	5.45	4.94	5.13
treatment	1	6.51	5.10	5.08	5.33	4.12
treatment	2	4.38	3.66	3.60	4.09	3.25
treatment	3	3.32	2.76	2.72	2.70	2.75
treatment	4	2.48	2.09	1.98	1.94	2.21
treatment	5	2.00	1.79	1.74	1.60	1.79
treatment	6	4.90	3.58	3.66	3.41	3.62
treatment	7	2.48	2.45	2.15	2.18	1.99
treatment	8	2.31	2.17	1.60	1.63	1.61

Dataset S3

Haematological and plsama composition data for uninstrumented shorthorn sculpin.

Blood was sampled and body mass and length were obtained after 5 days of experiment. The experiment consisted of 1 day in seawater (33 ppt) followed by 4 days of exposure to either seawater (33 ppt; control) or diluted seawater (15 ppt; treatment). The table show body mass

(B_M; g), length (mm), haemoglobin concentration (Hb; mg ml^{-1}), haematocrit (Hct; % red blood cells), potassium concentration (K; mmol l^{-1}), sodium concentration (N; mmol l^{-1}), chloride concentration (Cl; mmol l^{-1}), calcium concentration (Ca; mmol l^{-1}), osmolality (Osm; mOsm kg^{-1}) and mean corpuscular haemoglobin content (MCHC; g dl^{-1}).

Group	ID	B_M	Length	Hb	Hct	K	Na	Cl	Ca	Osm	MCHC
control	1	98.3	213	69	18	4.50	168.86	162.43	0.40	398	38.33
control	2	107.5	192	49.5	14	3.63	194.36	199.29	0.61	454.5	35.36
control	3	185	228	40	12.5	3.44	190.93	186.00	0.34	435	32.00
control	4	127.5	209	56.5	19	5.55	192.57	194.43	0.64	315.5	29.74
control	5	155	229	10.5	8	2.51	166.71	157.21	0.76	358	13.13
control	6	115.6	223	86.5	23.5	2.87	171.00	167.14	0.98	368.5	36.81
control	7	137.6	230	45	18.5	2.79	181.57	169.64	1.02	482.5	24.32
control	8	154.9	227	48	14.75	2.37	208.14	203.86	1.03	417	32.54
treatment	1	180	235	42	10	3.40	133.00	122.14	0.27	306	42.00
treatment	2	135.1	221	37	10.5	3.33	138.29	127.71	0.27	341	35.24
treatment	3	102.1	200	27	11	4.46	153.14	148.50	0.37	349	24.55
treatment	4	80.6	211	86	20	4.80	155.00	149.14	0.46	347.5	43.00
treatment	5	76.9	195	40	12.5	4.41	154.86	144.79	0.61	359	32.00
treatment	6	93.1	170	92.5	31	5.09	162.00	151.43	0.76	350.5	29.84
treatment	7	84.9	190	71	20.5	4.37	147.71	137.29	0.21	352.5	34.63
treatment	8	70.2	185	67	19.75	4.71	153.07	133.50	0.69	344	33.92

Dataset S4

Cardiorespiratory data for instrumented shorthorn sculpin.

Routine metabolic rate (RMR; mg O_2 h^{-1}), Cardiac output (CO; ml min^{-1}), Gut blood flow (GBF; ml min^{-1}), Heart rate (HR; beats min^{-1}), Stroke volume (SV; ml $beat^{-1}$), tissue oxygen extraction (TO_2; mg O_2 ml $blood^{-1}$), body mass (B_M; g) and length (mm) of sculpin surgically instrumented with blood flow probes either kept at their acclimation salinity (33 ppt; control) or short-term acclimated to diluted seawater (15 ppt; treatment).

Group	ID	RMR	CO	GBF	HR	SV	TO_2	B_M	Length
control	1	13.14	8.16	2.06	25.83	0.32	0.03	284.8	261
control	2	8.88	5.27	1.10	22.69	0.23	0.03	192.8	245
control	3	17.90	8.52		23.43	0.36	0.04	406	315
control	4	10.92	5.03	1.11	23.95	0.21	0.04	274.7	264
control	5	12.61	4.82	1.05	30.86	0.16	0.04	224.9	259
control	6	9.78	4.62		25.21	0.18	0.04	205.3	249
control	7	12.01	4.00	1.11	32.47	0.12	0.05	250.1	262
control	8	13.24	4.64	1.08	33.18	0.14	0.05	257.6	265
control	9	10.12	7.80	1.93	32.75	0.24	0.02	195.1	249
control	10	7.24	4.98	1.58	25.84	0.19	0.02	164.2	242
treatment	1	5.29	1.52	1.34	20.81	0.07	0.06	147.8	230
treatment	2	5.53	2.29	0.77	24.38	0.09	0.04	168.1	250
treatment	3	7.67	6.10	1.41	31.11	0.20	0.02	260	253
treatment	4	5.89	3.75	1.58	30.89	0.12	0.03	181.1	250
treatment	5	8.08	3.46	1.44	29.77	0.12	0.04	182.5	243
treatment	6	9.09	3.38	0.41	20.00	0.17	0.04	152.1	248
treatment	7	11.89	4.57	0.80	35.26	0.13	0.04	257.2	260
treatment	8	9.44	4.30	0.68	25.36	0.17	0.04	217.4	230
treatment	9	9.79	5.71	1.33	21.96	0.26	0.03	312.7	290
treatment	10	6.32	2.48		38.05	0.07	0.04	158.6	239
